# Oral Exposure to Atrazine Induces Oxidative Stress and Calcium Homeostasis Disruption in Spleen of Mice

**DOI:** 10.1155/2016/7978219

**Published:** 2016-11-10

**Authors:** Shuying Gao, Zhichun Wang, Chonghua Zhang, Liming Jia, Yang Zhang

**Affiliations:** ^1^Department of Toxicological Science, School of Public Health, Harbin Medical University, Harbin, Heilongjiang Province 150081, China; ^2^Harbin Center for Disease Control and Prevention, Harbin, Heilongjiang Province 150056, China; ^3^Environmental Monitoring Centre of Heilongjiang Province, Harbin, Heilongjiang Province 150056, China

## Abstract

The widely used herbicide atrazine (ATR) can cause many adverse effects including immunotoxicity, but the underlying mechanisms are not fully understood. The current study investigated the role of oxidative stress and calcium homeostasis in ATR-induced immunotoxicity in mice. ATR at doses of 0, 100, 200, or 400 mg/kg body weight was administered to Balb/c mice daily for 21 days by oral gavage. The studies performed 24 hr after the final exposure showed that ATR could induce the generation of reactive oxygen species in the spleen of the mice, increase the level of advanced oxidation protein product (AOPP) in the host serum, and cause the depletion of reduced glutathione in the serum, each in a dose-related manner. In addition, DNA damage was observed in isolated splenocytes as evidenced by increase in DNA comet tail formation. ATR exposure also caused increases in intracellular Ca^2+^ within splenocytes. Moreover, ATR treatment led to increased expression of genes for some antioxidant enzymes, such as* HO-1* and* Gpx1*, as well as increased expression of NF-*κ*B and Ref-1 proteins in the spleen. In conclusion, it appears that oxidative stress and disruptions in calcium homeostasis might play an important role in the induction of immunotoxicity in mice by ATR.

## 1. Introduction

**Figure 1:**
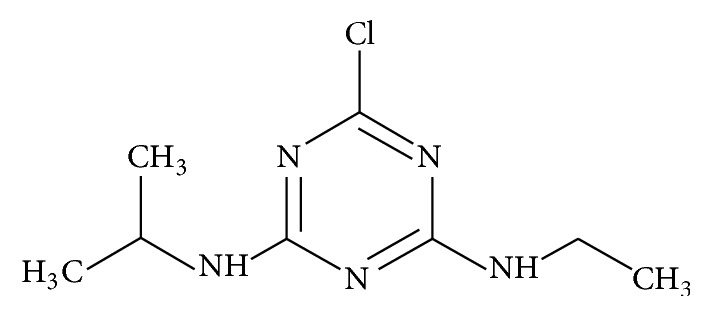
Structure of ATR.

Atrazine (ATR, 2-chloro-4-ethylamino-6-isopropylamino-*s*-triazine), a widely used broad spectrum herbicide, belongs to chloro-s-triazine family. Its chemical structure is shown in Figure 1. The primary mode of action of ATR in plants is to inhibit selectively the photosynthesis by specific binding to photosystem II, interrupting acyclic electron transport, decreasing CO_2_ assimilation. The blockage of photosynthetic electron flow may additionally result in the generation of high amounts of reactive oxygen species (ROS). The accumulation of ROS could cause consequent damage to cellular components of the plants [1]. The chemical characteristics of ATR include lipophilicity, low water solubility, slow hydrolysis, and high solubility in organic solvents [2]. ATR and/or its metabolites, such as deethylatrazine (DEA), deisopropylatrazine (DIA), and 2-hydroxyatrazine, are frequently detected in the environment, including in samples of soil, air, and water, and have even been measured in bodily excretions [3–5]. Humans can be exposed to ATR during manufacturing operations and by end-use applications such as farming and forestry [6]. Although ATR is considered a low toxicity herbicide, still, a number of toxicologic/epidemiologic studies have suggested that ATR could produce toxic consequences such as endocrine and reproductive alterations and behavioral and immunological dysfunction in animals and humans [7–10].

Immunosuppression caused by ATR is of great concern, as such dysfunction can foreshadow increases in risks for contracting diseases. There are many animal studies that have assessed the immunotoxic potential of ATR. Oral administration of ATR (at 250 and 500 mg/kg) for 14 days was immunotoxic in mice and was manifested by an increase in number of CD8^+^ T-cells and significant decreases in spleen cell numbers, spleen weight, and thymus weight [11, 12]. However, to date, the mechanism of action for this agent remains not fully understood. It has been suggested that chemicals can damage immune cells through a variety of biological mechanisms, including oxidative stress, alterations in calcium homeostasis, and apoptosis. In keeping with this premise, a previous study proved that Fas-mediated apoptosis was one mechanism for ATR toxicity among splenocytes of mice [13].

Oxidative damage, elicited primarily by increases in reactive oxygen species (ROS) generation, is involved in many deleterious biological processes. Many studies have suggested a link between oxidative stress and altered immune function in animals [14]. Excessive ROS formation can lead to lasting oxidative stress and can trigger many types of damage including changes in DNA and apoptosis. To combat deleterious effects of ROS, cells have evolved an intrinsic antioxidant defense network that consists of a glutathione redox system, comprised of reduced glutathione (GSH) and two important enzymes, glutathione reductase and glutathione peroxidase (Gpx1). Glutathione peroxidase-1 (Gpx1) is a pivotal intracellular enzyme that reduces hydrogen peroxide to water. Posttranscriptional regulation of Gpx1 expression during oxidative stress is via nuclear factor-*κ*B (NF-*κ*B) [15]. Heme oxygenase-1 (HO-1) is an inducible enzyme and a rate-limiting enzyme in heme degradation. Normally, HO-1 is found in very low levels in mammalian tissues [16] but is highly induced by a variety of stimuli, including oxidative stress. It has been shown that HO-1 can mediate antiapoptotic activities and help in the defense against oxidative stress in various types of cells [17]. Apart from damage to various cell constituents, ROS are also associated with dysregulation of intracellular calcium (Ca^2+^) homeostasis. Alterations of Ca^2+^ levels in cell cytoplasm brought about by ROS can serve as critical signaling components leading to the activation of the apoptotic pathway [18].

The current study was designed to evaluate potential alterations in ROS formation and in intracellular Ca^2+^ homeostasis as they pertain to any oxidative stress induced by host exposure to ATR. Apart from measures of those parameters, the studies also were designed to assess impact from these changes on endpoints that contribute to cell dysfunction, including changes in gene expression (associated with key molecules needed to mitigate oxidative stress) and damage to cell DNA. Taken together, it was expected that the data that would be gathered would greatly increase the understanding of mechanisms of immunotoxicity of ATR.

## 2. Material and Methods

### 2.1. Chemicals and Reagents

Atrazine (ATR, CAS# 1912-24-9; 98% purity) was obtained from ChemServices (West Chester, PA). Glutathione quantification kits were purchased from Jiancheng Bioengineering (Nanjing, China). Fura-2/AM was purchased from Life Technologies (Carlsbad, CA). Reverse transcriptase kits and FastStart Universal SYBR Green Master RT-PCR kits were obtained from Promega (Madison, WI). Antibodies against NF-*κ*B and Ref-1 host-/isotype-specific secondary antibodies were obtained from Santa Cruz Biotechnology (Santa Cruz, CA).

### 2.2. Animals

Male and female Balb/c mice (6–8-week-old) were purchased from Harbin Medical University Laboratory Animal Center (Harbin, China). Balb/c mice were selected because of their use in immunotoxicology studies and in our previous study in which splenocytes apoptosis induced by ATR was investigated [13, 19]. All mice were housed in specific pathogen-free facilities and maintained at 18–26°C with a 40–70% relative humidity and a 12 hr light-dark cycle. All mice had* ad libitum* access to standard rodent chow and filtered tap water. All procedures employed in this study were approved by Harbin Medical University Ethics Committee for animal research and were conducted in accordance with the Guide for the Care and Use of Laboratory Animals prepared by National Institutes of Health.

### 2.3. Treatments

After acclimatization for 1 wk, mice were assigned randomly to four groups (5 female and 5 male mice in each group). Because of its low water solubility, ATR was dissolved in 3% starch solution as a suspension [20]. For the animal treatments, ATR (100, 200, or 400 mg/kg body weight) in a suspension of starch was administered to groups of mice for 21 consecutive days by daily gavage (at volume of 20 mL/kg). Control animals received the starch solution vehicle. In the rationale for this dose regimen the following was considered: the acute (up to 14-day) oral median lethal dose (LD_50_) is 1332 mg/kg for mice [21]; the doses used in the 14-day NTP study were up to 500 mg/kg/day [11]; the doses were selected based on previous short-term ATR exposure study [13, 22].

### 2.4. Splenocyte Isolation

At 24 hr after the final dosing, each mouse was euthanized by cervical dislocation and their spleens were aseptically removed. The spleens of half the animals in each group were frozen immediately at −80°C and those of the remaining animals in each group were used for preparing splenocyte suspensions. In addition, blood was collected from all mice. After centrifugation (3000 ×g) for 10 min at 4°C, serum was isolated, aliquoted into Eppendorf tubes, and frozen at −80°C until used in analyses.

Single cell suspensions from each organ were then prepared in RPMI 1640 (Hyclone, Logan, UT) by passing the spleen through a 400 *μ*m pore size stainless steel mesh. The isolated cells were then washed with Hank's balanced salt solution (HBSS) and erythrocytes subsequently lysed by placing the cells in standard buffered lysing solution (0.15 M NH_4_Cl, 1 mM KHCO_3_, and 0.1 mM EDTA; pH 7.2) for 30 min at 37°C. After washing with HBSS, the cells were resuspended in RPMI 1640 and total cell levels/viabilities obtained using trypan blue. Following enumeration, cells were adjusted to different final concentrations in RPMI 1640.

### 2.5. Measurement of Intracellular ROS

Production of reactive oxygen in the cells was measured using redox-sensitive 2′,7′-dichlorodihydrofluorescein diacetate (DCFH-DA; Molecular Probes, Eugene, OR). DCFH-DA is nonfluorescent and readily diffuses across cell membranes and is then hydrolyzed by cellular esterases to nonfluorescent 2′,7′-dihydrodichlorofluorescein. The latter is converted to highly fluorescent 2′,7′-dichlorofluorescein (DCF) after reacting with intracellular ROS [23, 24]. Aliquots of splenocytes (total of 10^6^ cells) from each regimen were washed twice with phosphate-buffered saline (PBS, pH 7.4) and then incubated with 10 *μ*M DCFH-DA for 30 min at 37°C in the dark. Thereafter, the cells were washed twice again with PBS to remove excess probe. The mean fluorescence intensity (MFI) in 10,000 cells was then detected using a FACS flow cytometer (FACSAria, BD Biosciences, San Diego, CA) at excitation of 488 nm and an emission of 525 nm.

### 2.6. Measurements of Advanced Oxidation Protein Product (AOPP) Levels in Serum

Samples were prepared as follows: in a tube, 20 *μ*L of serum from each mouse was diluted into 100 *μ*L in PBS, followed by addition of 10 *μ*L of 1.16 M KI, and 20 *μ*L absolute acetic acid. The absorbance of the reaction mixture was immediately read in a SpectraMax 1601 spectrophotometer (Molecular Devices, Sunnyvale, CA) at 340 nm against a blank containing 100 *μ*L PBS, 20 *μ*L acetic acid, and 10 *μ*L KI solution. The linear range of chloramine-T absorbance at 340 nm was between 0 and 100 *μ*M. AOPP concentrations were expressed in *μ*M chloramine-T equivalents.

### 2.7. Glutathione Assay

The combined total (GSH_T_: reduced + oxidized forms), reduced, and oxidized (GSSG) forms of glutathione in serum were each determined using a glutathione quantification kit according to manufacturer instructions. In the assay, 5,5′-dithiobis-2-nitrobenzoic acid (DTNB; Ellman's reagent) reacts with reduced GSH to generate a colored product. Briefly, in 96-well plates, 20 *μ*L serum/mouse was combined with 150 *μ*L of reaction medium containing phosphate buffer (pH 7.2), DTNB, 1.5 mM NADPH, and 0.2 U/L glutathione reductase (GR). In reactions, to measure GSSG levels specifically, the GR was not present, thereby yielding only measures of levels of reduced GSH present; the difference in levels of total GSH after accounting for reduced GSH yielded the levels of GSSG. Each mixture was incubated in the dark for 60 min at room temperature before the optical density was measured using a microplate reader (Bio-Rad, Hercules, CA) at 412 nm. The GSH or GSH_T_ levels in each sample were then extrapolated from a standard curve prepared in parallel using GSH standard solutions.

### 2.8. Measurement of Splenocyte Intracellular [Ca^2+^]_i_ Levels

Splenocytes from each mouse were prepared to a density of 2 × 10^6^/mL in 10 mL complete RPMI 1640 medium (RPMI supplemented with 10% fetal bovine serum [FBS; Hyclone, Logan, UT], 2 mM L-glutamine, 2 mM sodium pyruvate, 100 U/mL penicillin, and 100 *μ*g/mL streptomycin, all from Sigma [MO, USA]). The splenocytes were then seeded into 6-well plates (at 2 mL/well) and treated with 5 *μ*M Fura-2/AM for 45 min at 37°C. Intracytoplasmic esterases hydrolyze Fura-2/AM to Fura-2 that, in turn, binds intracytoplasmic free Ca^2+^ to form Fura-2-Ca^2+^ complexes. Parallel cell suspensions used as blank controls had no Fura-2/AM loading. Thereafter, all cells were washed twice with complete RPMI 1640 and then underwent measures of fluorescence intensity [re: Fura-2] in an F-4600 fluorescence meter (Hitachi, Tokyo, Japan) at a 340 nm excitation wavelength and a 510 nm emission wavelength. From these measures, levels of [Ca^2+^]_i_ were determined and all data were expressed as *μ*M Ca^2+^.

### 2.9. Alkaline Comet Assays for DNA Damage

An alkaline comet assay was carried out as described previously [25]. All chemicals used in these assays were purchased from Sigma. In brief, isolated splenocytes (10^5^/sample) were mixed with 0.75% low-melting point agarose in PBS at 37°C and layered onto microscope slides precoated with 100 *μ*L normal-melting point agarose. Then the cells were dissolved for 40 min in a lysis buffer (pH 10.0; 2.5 M NaCl, 10 mM Tris, 100 mM Na_2_EDTA, 10% DMSO, 1% Triton-X). The slides were placed into a horizontal gel electrophoresis chamber and incubated in alkaline buffer solution (10 mM NaOH, 200 mM Na_2_EDTA, pH > 13) at room temperature for 20 min to facilitate DNA unwinding. This was immediately followed by electrophoresis at 20 V and 300 mA for 20 min. Slides were neutralized (0.4 M Tris, pH 7.5) three times (5 min intervals) and then stained with 5 *μ*g/mL ethidium bromide for fluorescence microscopy using a digital imaging system. Comets were visualized at 200x magnification. To quantify induced DNA damage, 100 randomly selected cells (50 cells from each of three replicate slides) were analyzed per sample with Tri Tek Comet Score Version 1.5 software (Warsaw, Poland). Parameters indicative of DNA damage included percentage DNA in the tail (% Tail DNA), tail length, tail moment, and Olive tail moment.

### 2.10. Extraction of Total RNA and Quantitative RT-PCR Analysis

The mRNA levels of* HO-1* and* Gpx1* in spleen tissues were quantified by real-time PCR. RNA from the frozen spleen samples was extracted using Trizol reagent (Invitrogen, Grand Island, NY) according to manufacturer instructions. Purity of the isolated material was confirmed using measures of absorbance at 260 and 280 nm. From 1 *μ*g of the resulting total RNA, cDNA products were synthesized using reverse transcriptase kits and then real-time PCR was performed with the FastStart Universal SYBR Green Master RT-PCR kit. Primers used in the analyses were synthesized by Invitrogen (sequences listed in Table 1). Thermal profiles were set as follows: 95°C for 10 min and 40 cycles of 95°C for 15 sec, 60°C for 30 sec, and 72°C for 30 sec. The values of the target genes and housekeeping gene (*β-actin*) were calculated for each sample using a standard curve. The standard curve for each gene was constructed from 5-fold serial dilutions of a single cDNA sample from an untreated control mouse, and the crossing points (CPs) were plotted against an arbitrary log concentration of each dilution. All target gene values were normalized to the level of *β-actin* in each sample.

### 2.11. Western Blot

Western blot analyses were performed to assess expression of NF-*κ*B and Ref-1. Spleen samples that were frozen were thawed and then homogenized in ice-cold 50 mM Tris-HCl buffer (pH 7.4) containing 0.25 M sucrose and a 1% protease inhibitor cocktail. The lysates were centrifuged (12,000 ×g) at 4°C for 10 min, and the protein content in the resulting supernatant was then determined by a Bradford protein assay. Equal amounts of protein were loaded into and then resolved over 10% (v/v) sodium dodecyl sulfate polyacrylamide gels by electrophoresis. The separated materials were then electrotransferred to a PVDF membrane (pore size, 0.22 *μ*m). Each membrane was blocked at room temperature for 1 hr in 5% nonfat milk (in Tris-buffered saline containing 0.1% Tween-20, TBST). Thereafter, each membrane was incubated with specific primary antibodies (diluted in TBST at 1 : 500 for anti-*β*-actin, 1 : 200 for anti-NF-*κ*B p65, and 1 : 200 for anti-Ref-1) for 2 hr at 37°C or overnight at 4°C with gentle shaking. After washing with TBST to remove unbound antibody, the membrane was then incubated with secondary antibody (diluted 1 : 2500 in TBST) for 2 hr at room temperature. After a final washing with TBST, the membrane was processed with ECL enhancing solution according to manufacturer protocols (Amersham, Piscataway, NJ). The intensity of the immunoreactive bands was then quantified by densitometry. In all cases, *β*-actin was used to normalize samples for loading.

### 2.12. Statistical Analysis

All data are reported as means ± SE. Statistical differences among treatment groups for each given endpoint were determined by one-way analysis of variance (ANOVA) followed by an LSD multiple-comparison test. A *p* value < 0.05 was considered significant. All the data were analyzed using SPSS v.17.0 software (SPSS, Chicago, IL).

## 3. Results

### 3.1. Generation of Splenocytes Intracellular ROS due to ATR

**Figure 2:**
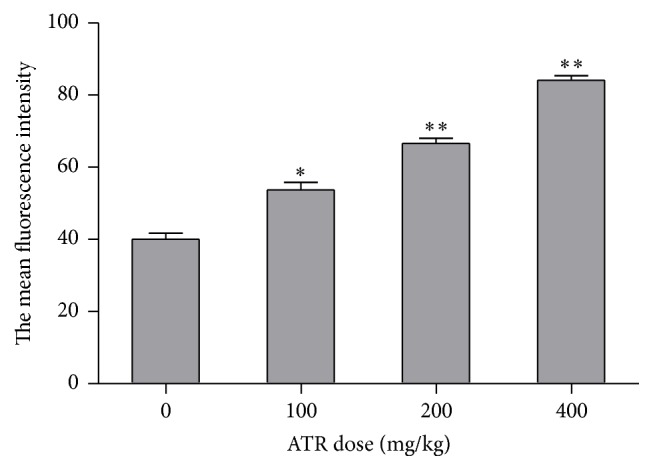
Flow cytometry analysis of ROS levels in splenocytes. Mice were exposed by daily oral gavage to ATR doses for 21 days. Cells were harvested 24 hr after the final dosing. Values shown are for means ± SE for five mice (males and females combined), analyzed individually. Value is significantly different from control (^*∗*^ *p* < 0.05; ^*∗∗*^ *p* < 0.01).

Analyses of intracellular ROS (Figure 2) indicated that formation of these products was upregulated in a dose-related manner as a result of each of the ATR treatments. Specifically, the mean fluorescence intensity of DCF in ATR treatment groups increased 1.34-, 1.66-, and 2.10-fold compared to the untreated group.

### 3.2. ATR Effects on Glutathione Levels and AOPP in the Serum

**Figure 3:**
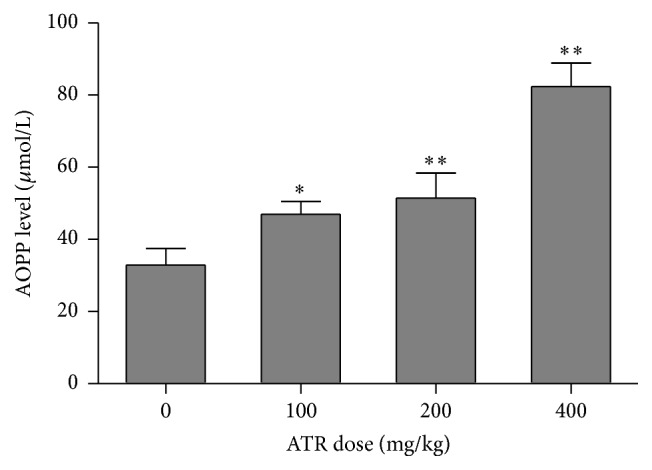
AOPP levels in serum. Mice were exposed by daily oral gavage to ATR doses for 21 days. Serum was harvested 24 hr after the final dosing. Values shown are for means ± SE for five mice (males and females combined), analyzed individually. Value is significantly different from control (^*∗*^ *p* < 0.05; ^*∗∗*^ *p* < 0.01).

The results of the analyses of total GSH indicated that there were significant decreases in levels of reduced (GSH_R_) and significant increases in oxidized (GSSG) glutathione in the serum of hosts treated with ATR (Table 2). With the highest ATR level tested, serum levels of GSH_R_ were to just 17% of control values (0.67 *μ*M versus 3.89 *μ*M), and the levels of GSSG increased 1.58-fold compared with the control (i.e., now 4.44 *μ*M from 2.81 *μ*M). Accordingly, GSH/GSSG ratios decreased significantly and in a dose-related manner, indicating oxidative stress in these cells. In concordance with those findings, serum levels of AOPP in mice treated with ATR were also significantly higher than in the sera of control mice. It increased 1.42-, 1.56-, and 2.50- fold in the ATR treatment groups, respectively, again in a dose-related manner (Figure 3).

### 3.3. ATR Effects on Intracellular [Ca^2+^]_i_ Levels in Splenocytes

**Figure 4:**
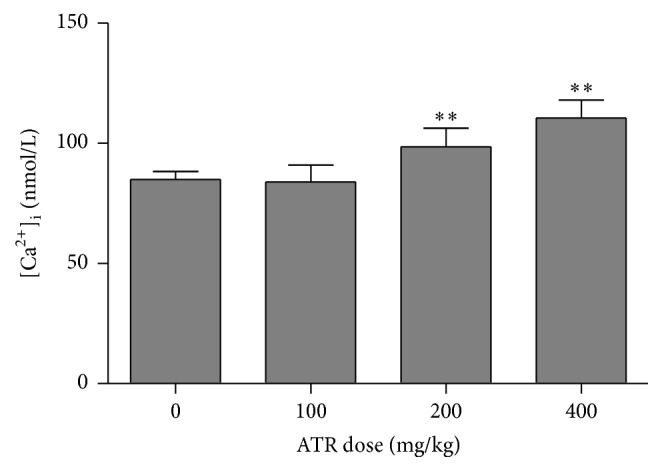
Intracellular [Ca^2+^]_i_ levels in splenocytes. Mice were exposed by daily oral gavage to ATR doses for 21 days. Cells were harvested 24 hr after the final dosing. Values shown are for means ± SE for five mice (males and females combined), analyzed individually. Value is significantly different from control (^*∗∗*^ *p* < 0.01).

Studies were done to assess if ATR treatment caused release of intracellular stored Ca^2+^ in the splenocytes of mice. The results indicated that intracellular Ca^2+^ increased in a dose-related manner due to ATR exposure (Figure 4). Specifically, concentrations of Ca^2+^ were significantly higher in the splenocytes of mice that received ATR at 200 (increase of 16%) or 400 (increase of 30%) mg/kg compared to levels in cells from control mice.

### 3.4. ATR Induction of DNA Damage in Splenocytes

**Figure 5:**
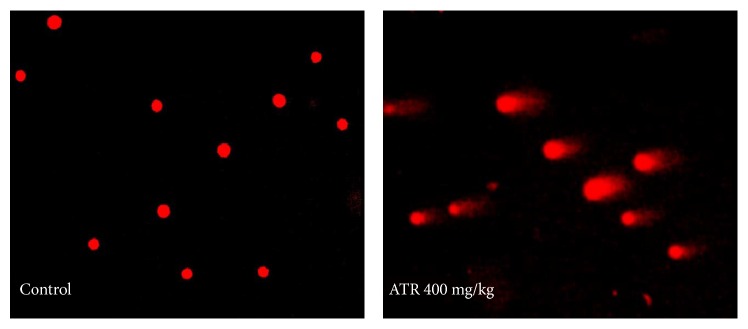
DNA damage in splenocytes. Mice were exposed by daily oral gavage to ATR doses for 21 days. Cells were harvested 24 hr after the final dosing. Representative images of ATR-induced DNA damage in splenocytes. Typical comets are shown in groups nontreated and treated with ATR (400 mg/kg). Magnification = 200x.

Table 3 presents results concerning levels of DNA damage in splenocytes of mice treated with different doses of ATR for 21 days.  Figure 5 presents a representative fluorescent figure used in the analyses. The % Tail DNA, tail moment, and Olive tail moment observed at each dose of ATR were all greater than those seen with cells from control mice. Specifically in the cells of mice in the 400 mg/kg group, statistically significant increases in % Tail DNA (12.47-fold increase), tail length (18.85-fold increase), tail moment (229.4-fold increase), and Olive tail moment (47.79-fold increase) were seen.

### 3.5. ATR Effects on* HO-1* and* Gpx1* Gene Expression

**Figure 6:**
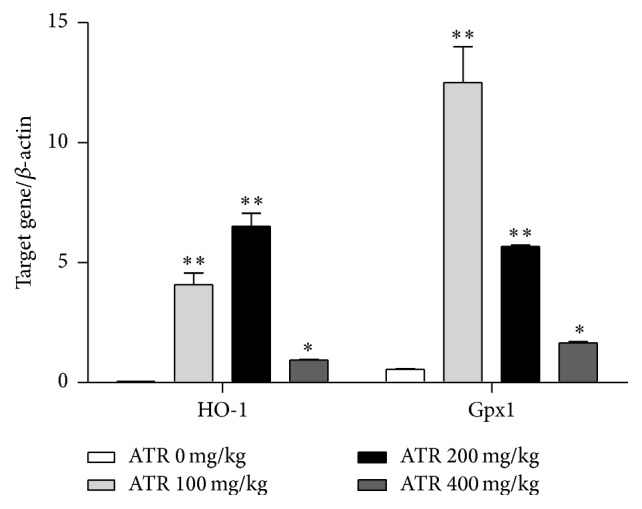
Effects of ATR on mRNA expression of splenic* HO-1* and* Gpx1*. Mice were exposed by daily oral gavage to ATR doses for 21 days. Tissues were harvested 24 hr after the final dosing. Data shown are representative of five independent experiments. In each independent experiment, RNA was extracted from one spleen of different mouse in each group. Results are expressed as means ± SE for five animals (males and females combined). Value is significantly different from control (^*∗*^ *p* < 0.05; ^*∗∗*^ *p* < 0.01).

The relative gene expression profiles of some genes in the spleen that are associated with responses to oxidative stress were examined by RT-qPCR. As shown in Figure 6, relative mRNA expression levels of* HO-1* and* Gpx1* were markedly increased as a result of exposure to all doses of ATR compared with values seen in the control mice, especially among spleens from mice in the 100 and 200 mg ATR/kg treatment groups. However, it should be noted that, for* HO-1*, the highest ATR dose led to a sharp decrease in expression. With* Gpx1*, the maximal effect on expression was obtained with 100 mg ATR/kg, with levels decreasing in a dose-trend manner thereafter, albeit still not reaching control values.

### 3.6. ATR Effects on NF-*κ*B and Ref-1 Protein Expression

**Figure 7:**
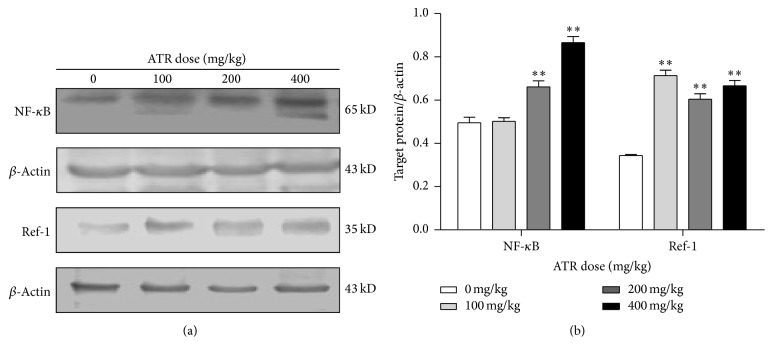
Effects of ATR on expression of NF-*κ*B and Ref-1 proteins in spleens. Mice were exposed by daily oral gavage to ATR doses for 21 days. Tissues were harvested 24 hr after the final dosing. Semiquantitative Western blotting was used to analyze expression of NF-*κ*B and Ref-1. Representative blots for NF-*κ*B and Ref-1 and respective blot for *β*-actin used for normalization are presented in (a). (b) Showing quantification results expressed as fold-change of the ratio of target protein/*β*-actin in each group. Data shown are means ± SE of three independent experiments. Asterisks illustrate statistically significant difference versus control (*p* < 0.01).

Expression of NF-*κ*B and Ref-1 in splenocytes was examined by Western blotting. The data showed that ATR treatment of the hosts induced 1.5- and 1.8-fold increases in the expression of NF-*κ*B at ATR doses of 200 and 400 mg/kg and 2.5–3.0-fold increases in Ref-1 expression (across all ATR doses tested) relative to expression levels in cells from control mice (Figure 7).

## 4. Discussion

To investigate potential mechanisms of immunotoxicity of ATR in mice, in particular the roles of oxidative stress and alterations in intracellular calcium homeostasis in the process, ROS production, DNA damage, intracellular Ca^2+^ concentration in splenocytes, as well as AOPP levels and GSH/GSSG ratios in serum, and expression of some key gene and proteins related to oxidative stress in the spleen were measured.

Excessive production of intracellular ROS/impaired function of cellular antioxidant systems can lead to oxidative damage. An aberrant increase in ROS levels might result in transient or permanent cellular alterations, including irreversible oxidative damage to DNA, programmed cell death, and/or cellular senescence [26, 27]. In a previous study from our laboratory, it was noted that ATR could induce lymphocytes apoptosis in the spleen at the same doses utilized in the present study [13].

Reduced glutathione (GSH) is a major component of the cellular antioxidant defense system. Damage to antioxidant capacity can result from overoxidation of GSH (or conversely a reduced ability to regenerate GSH from the GSSG form), as well as an overall reduction in intracellular concentrations of total (GSH + GSSG) glutathione [28]. In the present study, the levels of ROS in splenocytes were elevated; GSH depletion and a decrease in the GSH/GSSG ratio in the serum of the ATR-treated mice were also observed. This lack of GSH can not only be a product of the increases in ROS, but in turn could result in even further increase of ROS levels and toxicity [29] as the ROS are no longer being removed from the cell milieu. It was then not to be unexpected here that AOPP levels in serum were also increased in the ATR-treated mice. Various AOPP have been proposed as early markers of oxidative injury that originates as a result of actions of free radicals upon proteins [30].

Calcium is one of the most crucial intracellular messengers; this ion regulates cellular development, survival, and differentiation and is critical in the transition of a cell from state of reversible to irreversible cell death [31, 32]. Intracellular calcium concentrations must be precisely regulated as an increase in cytosolic Ca^2+^ concentration can activate intrinsic apoptotic and calcium-calmodulin kinase pathways [33]. In the current study, a marked increase in intracellular Ca^2+^ was noted with ATR doses of 200 and 400 mg/kg. It was seen in a previous study from this laboratory that the percentage of apoptotic cells increased in the spleens of mice after ATR exposure [13]. In many metazoan systems, Ca^2+^ overload is associated with pathological conditions, particularly in association with oxidative stress and subsequent cell death [34].

Many studies have documented that ATR can cause DNA damage* in vivo* and* in vitro* [35, 36]. In the present study, it was shown that ATR induced DNA damage in the splenocytes of exposed mice. The mechanism for this outcome is most likely an attack on DNA by induced increases in ROS formation; such toxicities can give rise to a variety of DNA lesions, including oxidized DNA bases, abasic sites, and DNA strand-breaks. In the end, this can and does lead to genomic instability.

To prevent oxidative stress, a cell must respond to ROS by mounting antioxidant defense systems including activation of many antioxidant enzymes.* Gpx1*, as a part of the GSH redox system, and HO-1 that catalyzes heme to biliverdin have long been considered cytoprotective against oxidative stress and in maintenance of cell homeostasis [37, 38]. Spleen expression profiles of genes encoding these two enzymes were investigated here to provide insight into potential mechanisms governing their activation in response to host ATR exposure. The results showed that expression of the* Gpx1* and* HO-1* genes was upregulated, especially with ATR at doses of 100 mg/kg, but then decreased. When pesticides lead to lipid peroxidation/oxidative stress, changes in antioxidant enzyme activities will be observed in tissue and blood samples; for example, overexpression of* Gpx1* and* HO-1* in tissues will offer protection from such pesticide-induced injury, but as these enzymes are increasingly utilized to detoxify free radicals, their activity will begin to decrease as was seen here [39].

Ref-1 is a multifunctional protein that not only regulates transcription factor activity but also mediates base excision repair. The transcriptional regulatory function of Ref-1 is mediated through its redox activity on several transcription factors including AP-1, p53, and NF-*κ*B [40]. In present study, expression of both Ref-1 and NF-*κ*B was increased significantly with all doses of ATR employed. NF-*κ*B is a redox-sensitive nuclear factor involved in the control of immune-inflammatory responses, developmental processes, and apoptosis. Some pesticides, like paraquat, have been shown to increase NF-*κ*B activity; this effect was accompanied by induced increases of ROS production [41]. That earlier finding was unsurprising in that it is well known that ROS can promote NF-*κ*B activation, proinflammatory cytokine formation, and apoptosis [27, 42]. Analyses of intracellular ROS here showed that formation of these products was upregulated in a dose-related manner as a result of the ATR treatments. As such, the current results with atrazine were in keeping with those seen with paraquat.

## Figures and Tables

**Table 1:** Primer sequences for RT-PCR assays.

Primer	Forward (5′-3′)	Reverse (5′-3′)	Size
*HO-1*	CACAGATGGCGTCACTTCG	GTGAGGACCCACTGGAGGA	130
*Gpx1*	GTGCAATCAGTTCGGACACC	CTTCTCACCATTCACTTCGCA	130
*β-Actin*	GTAAAGACCTCTATGCCAACA	GGACTCATCGTACTCCTGCT	227

**Table 2:** Effect of ATR on GSH and GSSG levels and GSH/GSSG ratios in serum.

ATR dose (mg/kg)	GSH (*μ*M)	GSSG (*μ*M)	GSH/GSSG ratio
0	3.89 ± 0.06	2.81 ± 0.05	1.39 ± 0.03
100	2.99 ± 0.16^*∗∗*^	3.20 ± 0.11^*∗∗*^	0.94 ± 0.06^*∗∗*^
200	1.70 ± 0.06^*∗∗*^	3.87 ± 0.11^*∗∗*^	0.44 ± 0.02^*∗∗*^
400	0.67 ± 0.04^*∗∗*^	4.44 ± 0.10^*∗∗*^	0.16 ± 0.02^*∗∗*^

Data are presented as mean ± SE (*n* = 5 per group).

^*∗∗*^ *p* < 0.01 versus control group (ATR = 0 mg/kg).

**Table 3:** Alkaline comet assay for DNA damage in splenocytes of mice.

ATR dose (mg/kg)	Tail DNA (%)	Tail length	Tail moment	Olive tail moment
0	3.57 ± 1.33	3.20 ± 0.51	0.12 ± 0.05	0.34 ± 0.13
100	9.27 ± 3.26	2.10 ± 0.53	0.32 ± 0.13	0.58 ± 0.19
200	18.28 ± 4.91^*∗*^	4.50 ± 1.59	1.24 ± 0.56	1.41 ± 0.44
400	44.53 ± 2.91^*∗*^	60.33 ± 3.88^*∗*^	27.53 ± 3.28^*∗*^	16.25 ± 1.65^*∗*^

Data are presented as mean ± SE (*n* = 5 per group).

^*∗*^ *p* < 0.05 versus control group (ATR = 0 mg/kg).

## Additional Points

*Summary*. The present data indicated that ATR could induce oxidative stress, DNA damage, disruption of calcium homeostasis, and alterations in the expression of select antioxidant enzymes in the spleens of mice. Thus, these results provide additional new insight into the mechanism of immunotoxicity induced by ATR.
